# Helical Polymer Working as a Chirality Amplifier to Generate and Modulate Multicolor Circularly Polarized Luminescence in Small Molecular Fluorophore/Polymer Composite Films

**DOI:** 10.1021/acscentsci.3c00122

**Published:** 2023-06-23

**Authors:** Shuo Ma, Biao Zhao, Jianping Deng

**Affiliations:** State Key Laboratory of Chemical Resource Engineering, College of materials Science and Engineering Beijing University of Chemical Technology, Beijing 100029, China

## Abstract

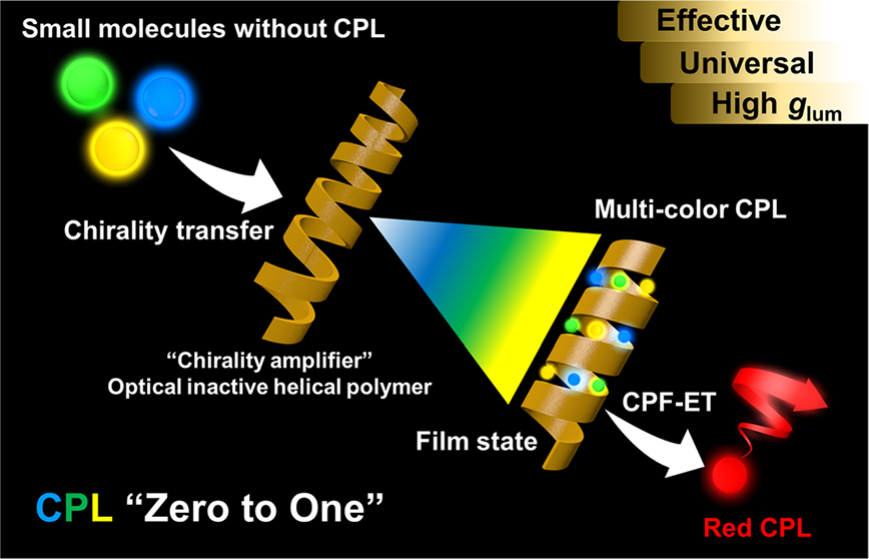

In-depth studies of chirality and circularly polarized luminescence (CPL) have become indispensable in the process of learning human nature. Small molecules with CPL activity are one of the research hotspots. However, the CPL properties of such materials are generally not satisfying. Here, we synthesized a series of chiral small molecular fluorophores that cannot demonstrate CPL emission themselves. By introducing an optically inactive helical polymer, chirality transfer and chirality amplification efficiently occur, thereby generating intense CPL emission. Through combining different chiralized fluorophores, multicolor CPL-active films with emission wavelength centered at 463, 525, and 556 nm were fabricated, with the maximum luminescence dissymmetry factor (*g*_lum_) being up to −0.028. Then, benefiting from the strong CPL emission and appropriate energy donor–acceptor system, we further established a circularly polarized fluorescence-energy transfer (CPF-ET) strategy in which the CPL-active films work as a donor emitting circularly polarized fluorescence to excite an achiral fluorophore (Nile red) as the acceptor, producing red CPL with *g*_lum_ of up to −0.011 at around 605 nm.

## Abstract 2

Multicolor circularly polarized luminescence emissions are achieved in small-molecule fluorophores/polymer composite films by taking optically inactive helical polymer as a chirality amplifier, with the highest luminescence dissymmetry factor of up to −0.028. By taking the obtained CPL-active films as donors, red CPL is further realized through a circularly polarized fluorescence-energy transfer strategy.

## Introduction

Chirality is a fundamental property widely existing in nature, and circularly polarized luminescence (CPL) reflects the chirality of luminescent substances in the excited state.^1−3^ Materials with CPL activity have attracted a large amount of attention due to their potential applications in the fields of information encryption, three-dimensional display, and bioimaging.^4−6^ Such materials are usually achieved by covalent bonding or chiral induction between chiral units and light-emitting units.^7^ Until now, CPL materials have been realized on the basis of rare earth complexes,^8^ organic small molecules,^9,10^ polymers,^11−13^ supramolecular self-assemblies,^14,15^ liquid crystal systems,^16,17^ and inorganic materials.^18,19^ Among them, organic small molecule materials have been widely studied due to their easily modified molecular structure, high luminescence properties, and definite structure–performance relationship.^20^ However, their CPL performances are generally modest, and not all chiral small molecules have CPL activity.

On the other hand, helical structures widely occur in nature and demonstrate inherent chirality, i.e., helical chirality, and have stimulated great interest in exploring artificial helical polymers.^21−23^ Compared with the chirality of small molecules, helical chirality tends to show a chirality amplification effect and easy regulation,^24,25^ so the artificial helical polymer-based CPL materials have promising and diverse CPL properties. CPL-active helical polymers have been effectively constructed by intramolecular chirality transfer or intermolecular chirality induction.^26−31^ Our team has prepared varieties of helical polyacetylene-based CPL materials through the copolymerization of chiral monomers and fluorescent monomers as well as the chiral induction of chiral helical polymers to fluorescent molecules.^32−35^ Inspired by the two types of materials, combining chiral small molecular fluorophores and helical polymers may not only improve the CPL performance of the former but also solve the limitation of chiral polymers with expensive monomers and limited types from the perspective of achiral polymers. Unfortunately, such an interesting and promising research topic still remains to be explored. The present work reports our recent success in judiciously combing chiral small molecular fluorophores with optically inactive helical polymer: the former simultaneously provides chirality and fluorescence but without CPL performance, while the latter serves as a chirality amplifier; the synergistic effects between the two components produce intense and multicolor CPL emission.

Fluorescence resonance energy transfer (FRET) can realize the transfer of fluorescence (FL) energy between different luminescent molecules.^36,37^ On this basis, circularly polarized fluorescence resonance energy transfer was proposed and studied by scientists.^38,39^ In this concept, besides FL energy transfer, chirality transfer occurring between donors and acceptors is usually indispensable. Zhu et al. synthesized a cholesteric unit containing tetraphenylethylene luminescence structure and realized the transformation from blue color CPL to yellow color CPL with circularly polarized fluorescence resonance energy transfer after coassembly with achiral luminescence acceptor. Unfortunately, when the donor and acceptor are separated from each other, the system can realize only fluorescence energy transfer but fail in transfer chirality.^38^ To solve the problem, radiative energy transfer can come into play.^40,41^ Most recently, we achieved a circularly polarized fluorescence energy transfer (CPF-ET) strategy and obtained multicolor CPL emissions, in which both radiative and nonradiative energy transfer modes were adopted.^42^

**Scheme 1:**
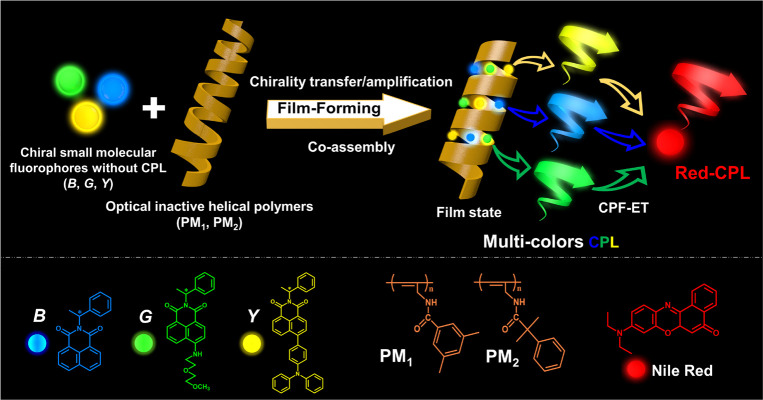
Illustration of the Preparation Process for Multicolor CPL and CPF-ET

In the present work, we prepared 1,8-naphthalimide-derived chiral fluorophores using chiral phenylethylamine enantiomers, obtaining blue, green, and yellow chiral small molecular fluorophores, although with molecular chirality and FL emission the luminophores show no CPL activity. However, as shown in Scheme 1, we blend them with optically inactive helical polyacetylenes to prepare chiral fluorescent casting films. During film forming, the chirality of chiral small molecular fluorophores can be effectively transferred to polyacetylenes by noncovalent interaction and significantly amplified, and a certain degree of coassembly occurs, finally achieving CPL performance out of nothing. This strategy innovatively complements the shortcomings of the two types of materials in this material system, which can effectively prepare CPL-active films and is conducive to achieving a high luminous asymmetry factor and practical applications. In addition, the FL emission of three-colored chiral small molecular fluorophores and the absorption of Nile red dye conform to the donor–acceptor (D–A) energy relationship. That is, the photons released by CPL-active films can be effectively absorbed by the Nile red dye and used for new transitions. Hence, through circularly polarized fluorescence energy transfer (CPF-ET), we further used the prepared three-colored CPL-active films as donors to excite Nile red and achieve red-color CPL emission. The work offers a new alternative for taking advantage of the synergistic effect between chiral small molecular fluorophores and optically inactive helical polymers, in which each constituent makes a unique contribution to accomplishing intense CPL emissions.

## Results and Discussion

### Photophysical and Chiroptical Properties of Chiral Small Molecular Fluorophores

**Figure 1:**
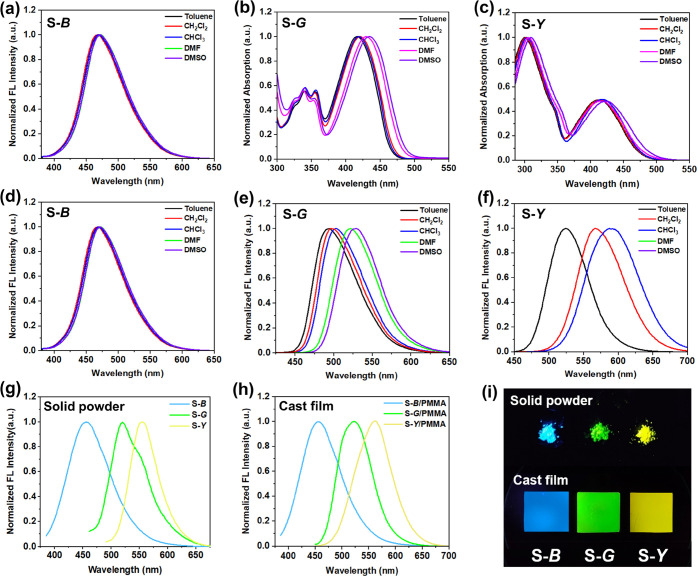
(a–c) Absorption spectra and (d–f) FL emission spectra of S-*B*, S-*G*, and S-*Y* in different solvents (*c* = 0.1 mg/mL). FL emission spectra of S-*B*, S-*G*, and S-*Y* in (g) the solid powder state and (h) the cast film state. (i) Photographs of S-*B*, S-*G*, and S-*Y* in solid powder and cast films under UV light (λ_ex_ = 365 nm).

We synthesized three chiral 1,8-naphthalimide derivatives (Scheme 1), defined as S/R-*B*, S/R-*G*, and S/R-*Y*, respectively.^43^ Detailed synthesis steps and structural characterizations are presented in the Supporting Information (SI) (Figures S1–S3). As shown in Figure 1a and 1d, the absorption and emission spectra of S-*B* in different solvents are very stable, without an obvious difference. However, with the increase in solvent polarity, both *G* and *Y* containing D–A structure show a slight red shift in absorption spectra and a significant red shift in FL emission spectra (Figure 1b,c,e,f and Figure S4), which is consistent with the characteristics of intramolecular charge transfer (ICT).^44,45^ Besides, the FL emission of S-*Y* is quenched in strong polar solvents, i.e., *N*,*N*-dimethylformamide (DMF) and dimethyl sulfoxide (DMSO), which may be due to the formation of the hydrogen bond interaction between S-*Y* and strongly polar solvent molecules, resulting in reduced energy levels of excited states, enhanced intersystem crossing, and internal conversion. Consequently, S-*Y* molecules undergo nonradiative transition, leading to the serious decrease in the fluorescence quantum yield or even fluorescence quenching.^46,47^ ICT can effectively regulate the FL emission color of chiral small molecular fluorophores and obtain higher emission efficiency. Under UV irradiation, S-*B*, S-*G*, and S-*Y* show obviously visible blue, green, and yellow emission in the powder state and the poly(methyl methacrylate) (PMMA) casting film state (Figure 1g–i), with quantum yields (Φ) of 19.9, 66.3, and 47.4%, respectively. Embedding chiral small molecular fluorophores into the polymer matrix can effectively reduce the interference of external factors and contribute to a more stable photoluminescence property.

**Figure 2:**
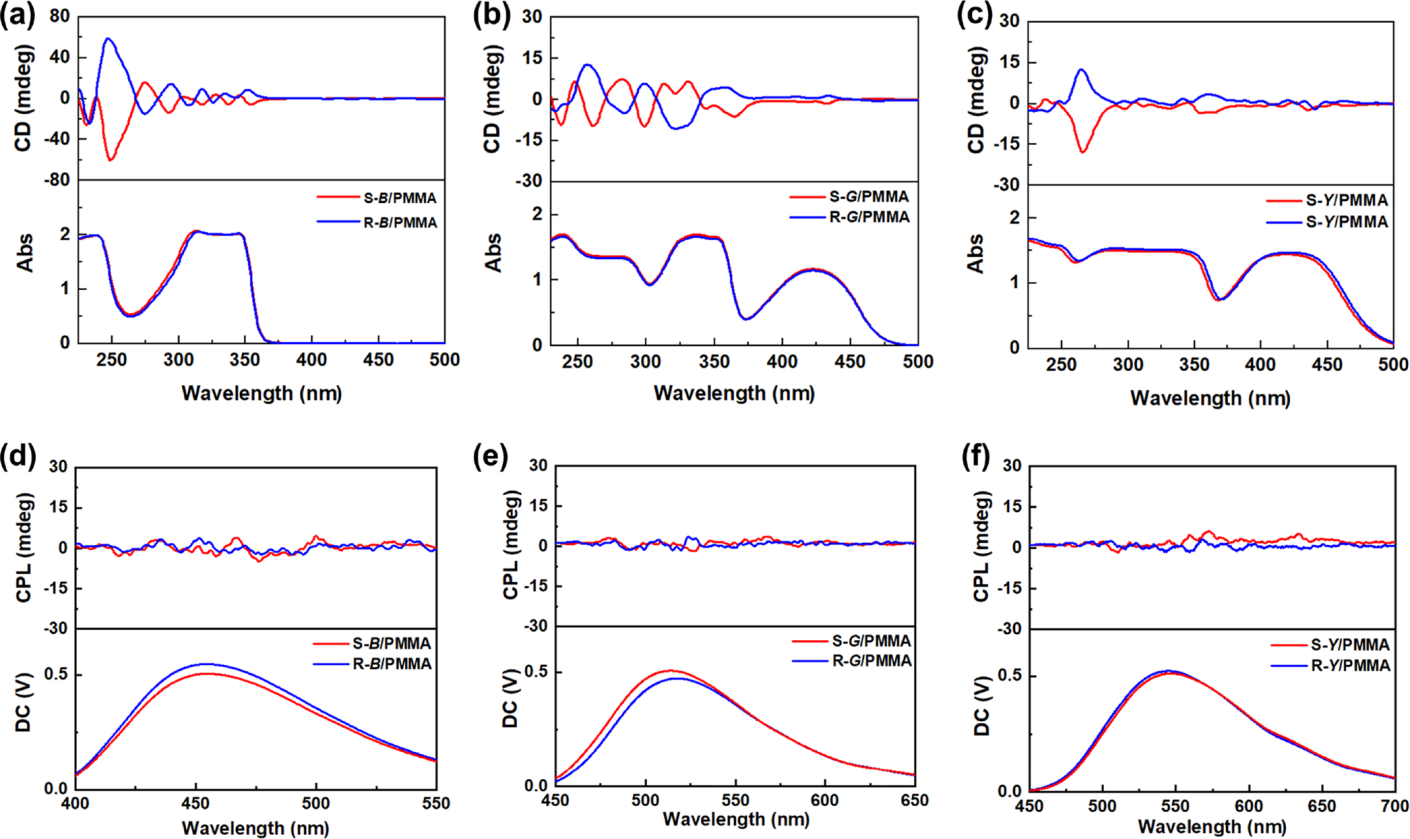
(a–c) CD and (d–f) CPL spectra of S/R-*B*, S/R-*G*, and S/R-*Y* in PMMA cast films.

Chiroptical properties of the prepared chiral small molecular fluorophores were studied in a trichloromethane (CHCl_3_) dilute solution and PMMA cast film. As shown in Figure S5, these chiral small molecular fluorophores show only a slight Cotton effect in the solution state. However, they have more significant Cotton effects in the wavelength range of 250–350 nm in PMMA films and exhibit opposite signals for S and R enantiomers (Figure 2a–c). These Cotton effects originate from the one-handed supramolecular tilt chirality based on the aromatic packing of chiral small molecular fluorophores.^48^ Considering that these chiral small molecular fluorophores contain luminescent structures and chiral centers, we further characterized their CPL performance. However, unfortunately, all of the chiral fluorescent molecules failed to show any CPL-active emission in both dilute solution and PMMA films (Figure S6 and Figure 2d–f) probably because their chirality is too weak or not successfully transferred to the luminescent structure on the molecules.

### Amplification of Chirality and Generation of Multicolor CPL Emissions

From small molecules to the corresponding helical polymers, the chirality is usually effectively amplified. Therefore, helical polymers have been widely used in the construction of CPL materials. Among them, it is more interesting to use a chiral polymerization environment or chiral medium postinduction to realize the preparation of chiral polymers with CPL active from achiral monomers/achiral polymers,^26−28,33,49^ which can effectively avoid the limitation of expensive chiral substances with limited variety on the development of more novel materials. Our team has constructed various CPL-active materials based on helical polyacetylenes.^32,34,35,50,51^ Herein, we anticipate achieving CPL emission from the chiral small molecular fluorophores by means of chirality transfer and chiral amplification contributed by optically inactive helical polyacetylene. This idea can solve some inherent shortcomings of chiral small molecular fluorophores and optically inactive helical polymers; namely, we can take advantage of the synergistic effects between them. For this purpose, an achiral substituted acetylenic monomer (M_1_) was synthesized and polymerized for the subsequent research (for the molecular structure of M_1_ and its polymer PM_1_, refer to Scheme 1). The NMR spectra demonstrate the successful preparation of the monomer (Figure S7). Then, FT-IR spectra of M_1_ and PM_1_ were recorded. As clearly shown in Figure S8, the characteristic peak (2120 cm^–1^) belonging to the C≡C bond disappeared after the reaction, indicating the successful polymerization of M_1_. Due to the alternating structure of C–C and C=C, substituted polyacetylene has two configurations, *cis* and *trans*, and their relative content directly affects the stereoregularity of the molecular chain of substituted polyacetylene. Here, we characterized PM_1_ by Raman spectroscopy and found that its *cis* content is as high as 88% (Figure S9), indicating a high degree of stereoregularity, which is conducive to the formation of a helical backbone structure. Furthermore, no CD signal was detected in the CD spectrum of PM_1_, but a typical absorption peak (350 nm) appeared in the UV–vis spectrum, demonstrating that PM_1_ is optically inactive (Figure S10).

**Figure 3:**
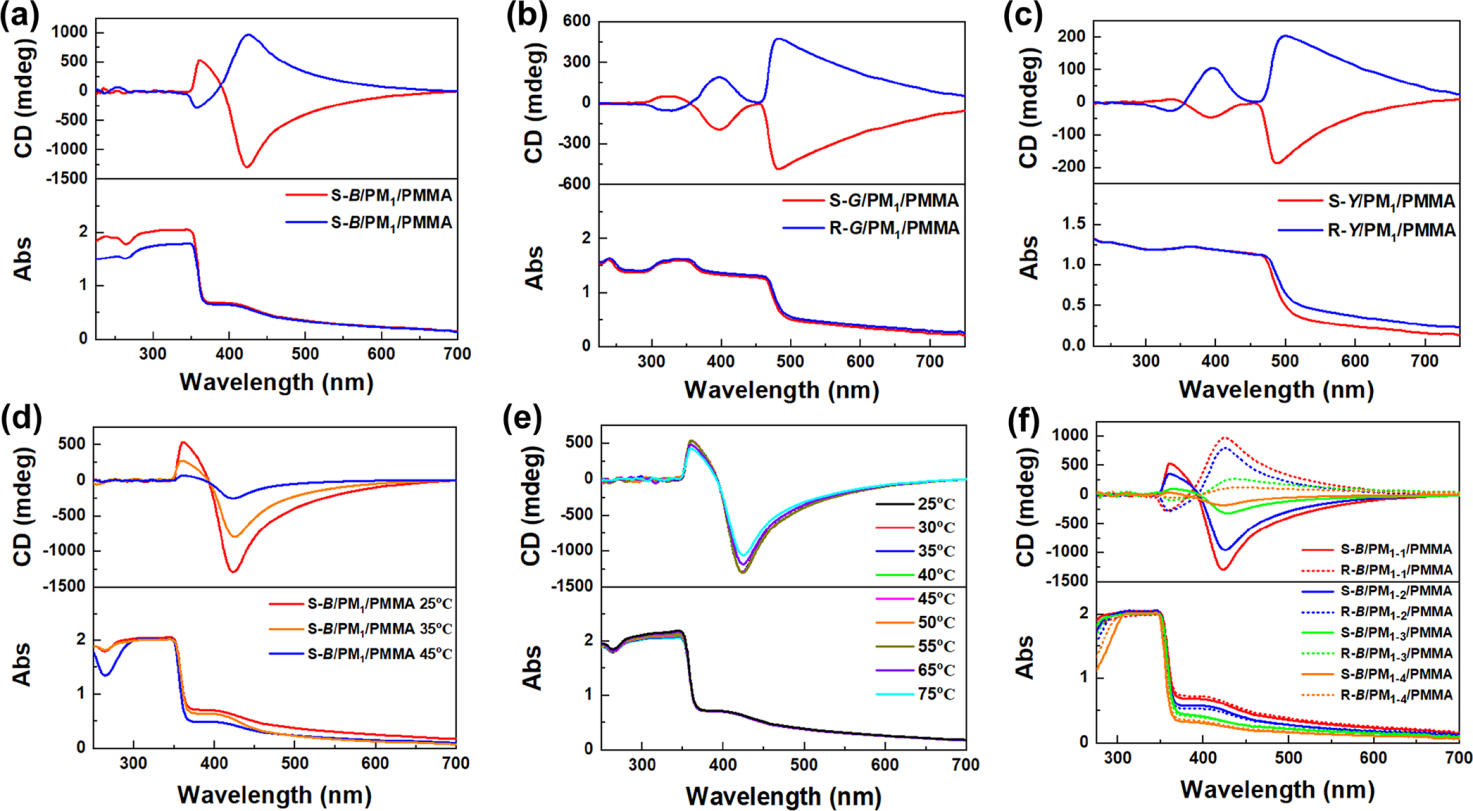
CD and UV–vis spectra of (a–c) the small molecule/polymer composite films, (d) S-*B*/PM_1_/PMMA films prepared at different film-forming temperatures, (e) the S-*B*/PM_1_/PMMA film (fabricated at 25 °C) measured at different temperatures, and (f) the S-*B*/PM_1_/PMMA film with different molecular weights of PM_1_.

PM_1_ and chiral small molecular fluorophores were dissolved in the CHCl_3_ solution of PMMA, and multicomponent composite films were fabricated by casting. Excitingly, all of the chiral fluorescent films added with PM_1_ show far stronger optical activity than the small molecules themselves in the film state, with effective amplification by tens of times (Figure 3a–c). These results indicate that the chirality of chiral small molecular fluorophores was successfully transferred to optically inactive helical polyacetylene and significantly amplified. The CD signals of S/R-*B*/PM_1_/PMMA films are slightly different from those of the other films, but all of the CD signals at lower wavelength (<400 nm) come from the induced one-handed helical structure of PM_1_^34,35^ while the CD signals at higher wavelength (>400 nm) belong to a typical molecular assembly phenomenon.^11,52,53^ The optical activity of the three chiral fluorescent films decreases regularly from *B* to *Y*, which is contrary to the steric hindrance of the substituent in the chiral small molecules. Thus, the difference in the overall optical activity of the materials may be attributed to the molecular structure of the chiral small molecular fluorophores and their interactions with helical polyacetylene. Besides, according to the CD spectra in Figure 2a–c and Figure 3a–c, we further calculated the absorption dissymmetry factor (*g*_abs_) of chiral small molecular fluorophores and the composite films, as presented in Table S1. Compared with the small molecules, the *g*_abs_ values of the prepared composite films were significantly amplified just like the CD results, further confirming the chirality amplification effect.

It is worth mentioning that the mixed solutions before film formation did not show a regular Cotton effect (Figure S11), so the above chirality amplification effect occurred during the film-forming process. FL emission of the composite films had no significant difference from the corresponding one prior to film formation, indicating that the photoluminescence property of the luminophores was not affected (Figure S12). We also observed the internal structure of films with an optical microscope. As shown in Figure S13, compared with the pure PMMA film without other components, the composite films are not uniform at the microscopic level and different degrees of aggregation occurred. Thereinto, the S-*B*/PM_1_/PMMA film has the most obvious aggregation, the S-*Y*/PM_1_/PMMA film shows the weakest aggregation, and the aggregation degree seems to be related to the optical activity intensity of the films. Furthermore, the CD and UV–vis spectra of the S-*B*/PM_1_/PMMA film remain almost identical at different test angles (Figure S14), so the CD signals from prepared films are generated from the inherent optical activity of the films rather than the interference of linear polarization.

Besides, taking S-*B*/PM_1_/PMMA films as an example, the magnitude of CD decreased significantly at higher film-forming temperatures (Figure 3d). The peak of the CD signal for the S-*B*/PM_1_/PMMA film prepared at 45 °C is only about 20% of that at 25 °C; namely, the optical activity of films is quite sensitive to the film-forming temperature. The decrease in the overall optical activity indicates that the increase in temperature affects both the chiral induction effect of fluorescent small molecules toward PM_1_ and the assembly effect of (macro)molecules. This result is reasonable because chiral induction and molecular assembly normally rely on noncovalent interaction forces between different components.^47,54−56^ For this reason, we took the S-*B*/PM_1_ system as an example and recorded the FT-IR spectra to explore the inherent factors promoting chirality transfer in composite films. As shown in Figure S15, the N–H bond on the amide group of PM_1_ exhibits a stretching vibration peak at 3475 cm^–1^. When PM_1_ was mixed with S-*B*, the characteristic peak shifted to low frequency by about 45 cm^–1^. And this phenomenon also occurs in composite films. Meanwhile, the stretching vibration peak (1708 cm^–1^) of the carbonyl unit (−C=O) in S-*B* also slightly shifts to low frequency after mixing the two components. This indicates that the N–H unit in PM_1_ may form hydrogen bonds with the −C=O unit in S-*B*, and the hydrogen bond interaction may be the key to achieving the successful chirality transfer from chiral small molecular fluorophores to optically inactive helical polymer. Besides, the prepared films have a certain thermal stability, causing the CD signals at different test temperatures to change only slightly (Figure 3e). Even at 75 °C, the CD signals remain nearly constant when compared with that measured in a room-temperature environment. This further indicates that the enormous change in optical activity of the films is caused by the influence of temperature on the chirality induction and assembly behaviors in the film formation process rather than the influence after film formation, such as the temperature sensitivity of helical polyacetylene. In addition, we synthesized a series of PM_1_ with different molecular weight to explore the effect of molecular weight on the optical activity of the composite films. As shown in Figure S16, the molecular weight of the prepared PM_1_ varies from 3480 to 5180, and there is no significant change in the polydispersity. When these polymers were doped into the composite films, it is found that the optical activity of the composite films decreases with the reduction of molecular weight (Figure 3f), suggesting that the chirality amplification effect in composite films can be regulated by changing the molecular weight of helical polyacetylene.

**Figure 4:**
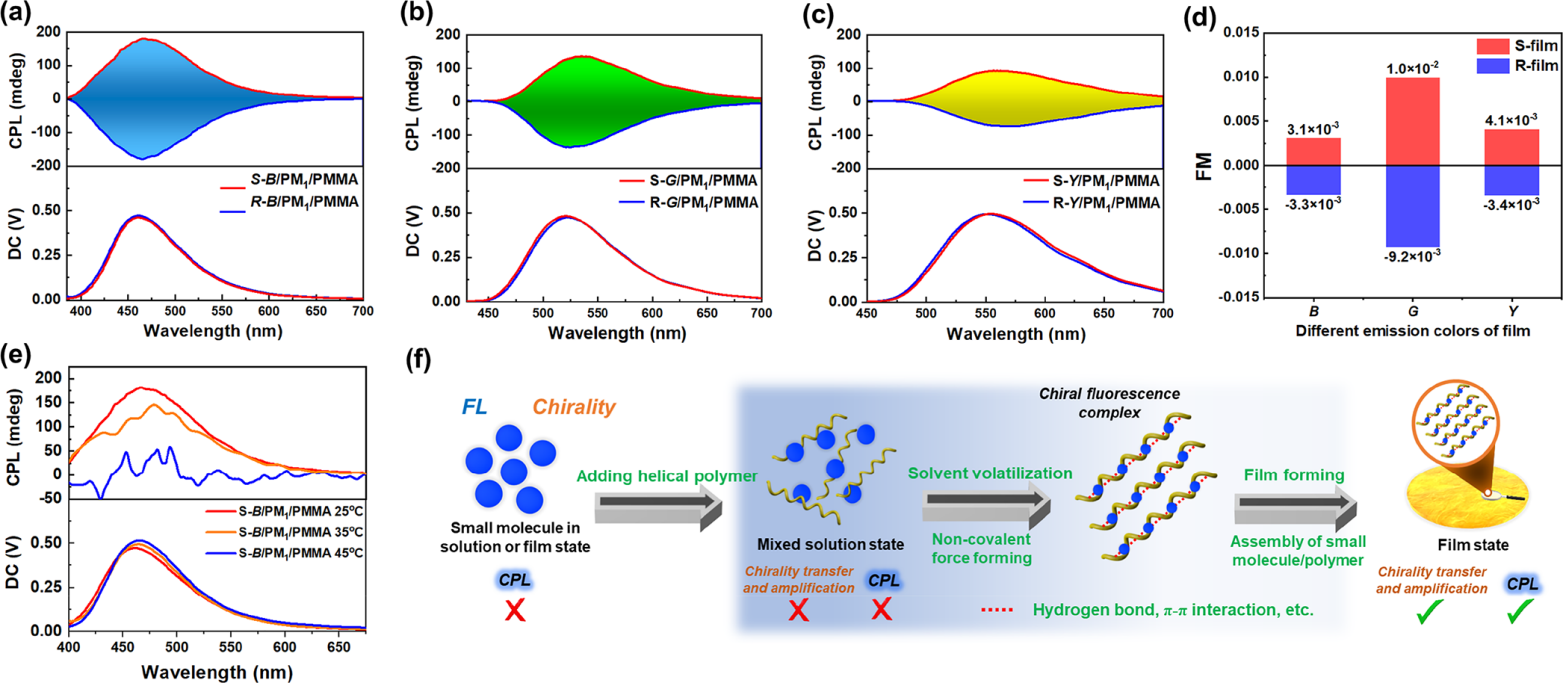
(a–c) CPL spectra and (d) FM factors of the three-color CPL-active films (λ_ex_ = 365 nm, slit width = 3000 μm). (e) CPL spectra of S-*B*/PM_1_/PMMA films prepared at different film-forming temperatures. (f) Proposed mechanisms for chirality transfer and amplification and CPL generation in the composite films.

Then, the CPL performance of composite films doped with chiral small fluorophores and PM_1_ was characterized. As we expected, all films show excellent CPL emission due to their significant optical activity (Figure 4a). The S-composite films exhibit intense positive CPL emissions, and the R-composite films show negative CPL emissions. The luminescence dissymmetry factor is one of the important indicators to evaluate the CPL performance, defined as *g*_lum_ = 2 × (*I*_L_ – *I*_R_)/(*I*_L_ + *I*_R_), where *I*_L_ and *I*_R_ represent the luminescence intensities of left and right circularly polarized light, respectively. The calculated *g*_lum_ values are on the order of the 10^–2^ level, as summarized in Table 1. Besides, we also introduced the concept of the “figure of merit” (FM)^7^ to evaluate the dissymmetry degree and emission intensity of CPL performance more intuitively, which is defined as FM = *g*_lum_ × Φ. As shown in Table 1, among the different CPL films, S(R)-*G*/PM_1_/PMMA films have the largest FM values.

**Table 1:** Summary of the CPL Data in the Small Molecule Fluorophore/Polymer Composite Films

	S-*B*/PM_1_/PMMA	R-*B*/PM_1_/PMMA	S-*G*/PM_1_/PMMA	R-*G*/PM_1_/PMMA	S-*Y*/PM_1_/PMMA	R-*Y*/PM_1_/PMMA
Wavelength	466 nm	469 nm	534 nm	526 nm	562 nm	569 nm
*g*_lum_	2.7 × 10^–2^	–2.8 × 10^–2^	2.1 × 10^–2^	–1.9 × 10^–2^	1.3 × 10^–2^	–1.1 × 10^–2^
Φ (%)	11.5	11.8	49.4	48.3	31.6	30.6
FM	3.1 × 10^–3^	–3.3 × 10^–3^	1.0 × 10^–2^	–9.2 × 10^–3^	4.1 × 10^–3^	–3.4 × 10^–3^

Therefore, we successfully used the chirality induction between chiral small molecular fluorophores and optically inactive helical polyacetylene and the film-forming self-assembly to realize the preparation of multicolor CPL-active films. Compared with the reported small-molecule-based CPL materials, the CPL magnitude in this study is appreciable, demonstrating that our CPL material design strategy is feasible and efficient. The CPL intensity also decreased significantly with the increase in the film-forming temperature (Figure 4e), which is consistent with the CD signals, further indicating that the optical activity of materials directly affects the CPL performance. The high temperature will affect the chirality induction effect, resulting in lower CPL activity. We speculate that the mechanism of chirality induction and CPL generation in this study is as follows. In the CHCl_3_ solution of the chiral small molecular fluorophores and optically inactive helical polyacetylene, it is difficult for them to directly form a strong noncovalent interaction to achieve chirality transfer due to their relatively low concentrations. Then, the film-forming matrix (PMMA) is added and the solvent is volatilized under a constant temperature. With the decrease in solvent, hydrogen bonds and π–π and CH−π interactions are gradually formed between the chiral small molecular fluorophores and optically inactive helical polyacetylene, and the two can be regarded as a whole chiral fluorescent complex. At the same time, the difference in compatibility between this chiral fluorescent complex and the PMMA matrix will lead to microscopic molecular arrangement and aggregation, thus giving the material high-level chirality. Finally, with the help of the one-handed polymer helical structure induced by chiral small molecular fluorophores and the molecular assembly process, the optical activity of chiral small molecular fluorophores is greatly amplified. Combined with their photoluminescence properties, the chiral fluorescent complex exhibits intense intrinsic CPL emission in the film state. The entire process discussed above is summarized in Figure 4f.

**Figure 5:**
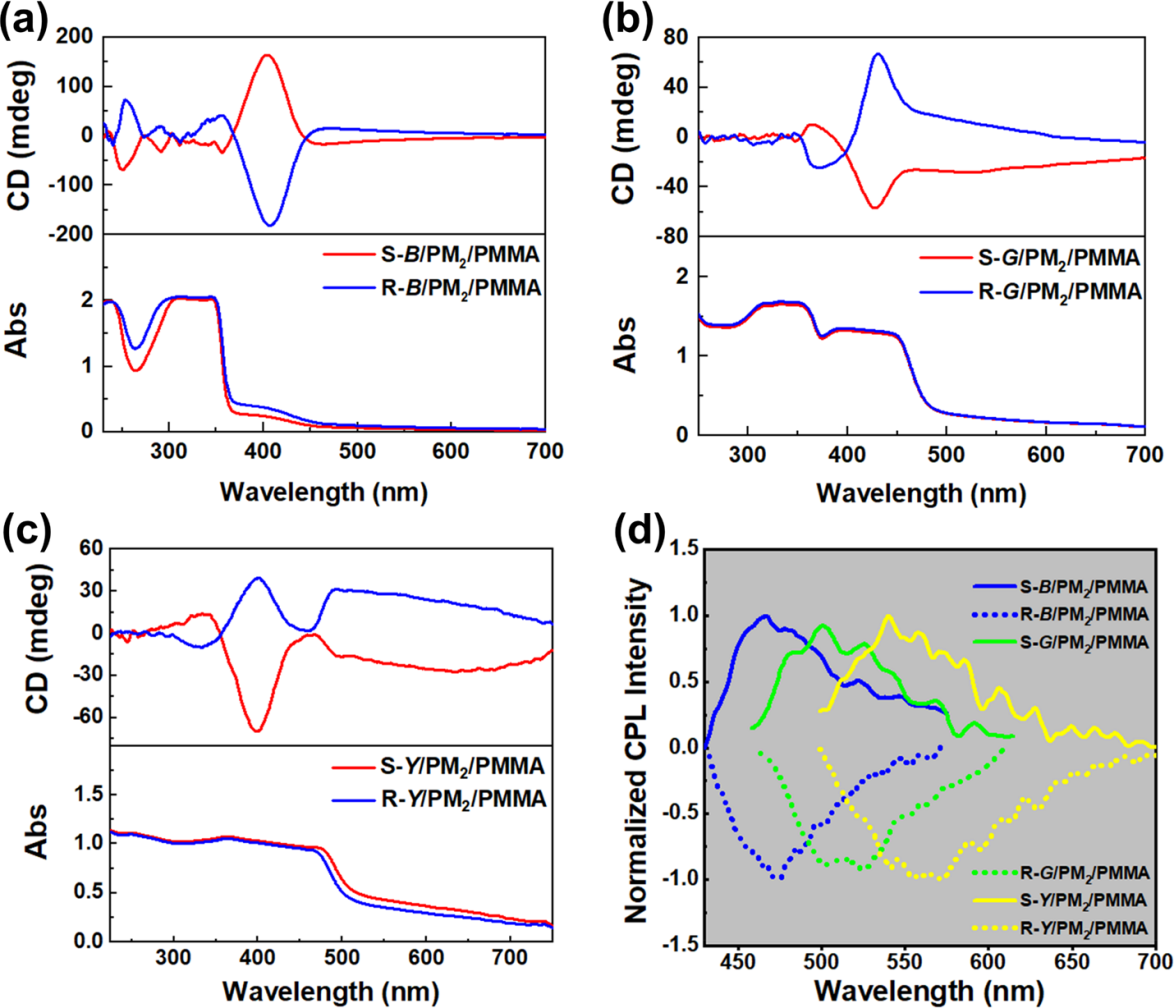
(a–c) CD and (d) CPL spectra of the CPL-active films based on PM_2_ (λ_ex_ = 365 nm, slit width = 3000 μm).

To further highlight the CPL design strategy, we synthesized another optically inactive helical polyacetylene (defined as PM_2_, Scheme 1) with a *cis* conformation of 83% (Figures S17–S19). Then, PM_2_ was used to replace PM_1_ to prepare the other chiral fluorescent composite films with the same chiral small molecular fluorophores. As shown in Figure 5a–c, all of the films also show obvious optical activity, and the peak intensity of the CD signal is consistent with that of the films doped with PM_1_, indicating that our aforementioned demonstration of this phenomenon is reasonable. Furthermore, all of the chiral fluorescent films emit remarkable mirror-imaged CPL signals (Figure 5d). The films prepared with the S-luminophore complex show positive CPL signals, while the films prepared with the R-luminophore complex show negative CPL signals. And the *g*_lum_ of films doped with S/R-*B*, S/R-*G*, and S/R-*Y* are 2.8 × 10^–3^/–3.0 × 10^–3^, 1.8 × 10^–3^/–1.3 × 10^–3^, and 1.2 × 10^–3^/–1.0 × 10^–3^, respectively. The difference in the molecular structure of PM_1_ and PM_2_ may be the main reason for the difference in the chiroptical performance of the prepared composite films. Here, the chirality induction from chiral small molecular fluorophores to an optically inactive helical polymer mainly contains two steps: (1) Intermolecular interactions including hydrogen bonding and the π–π effect are first formed within the amide group and benzene ring unit of polyacetylene and fluorophore, endowing the formation of the pseudochiral center in the pendant of polyacetylene. (2) The induced pseudochiral center further transfers its chirality to the main-chain backbone of polyacetylene during film formation, yielding the prepared composite film with intense optical activity. Compared with PM_2_, the benzene ring unit in PM_1_ is closer to the main-chain backbone, which makes the chiral induction process more efficient in PM_1_. As a consequence, the chiroptical performance in PM_1_-based composite films is better than that in PM_2_-based composite films. Based on the above results, it can be found that our proposed design strategy for CPL materials is reasonable and effective. Following this strategy, the CPL performance of small molecular materials can be improved and more novel CPL materials will be developed, i.e., by making full use of helical polymers as chiral amplifiers.

### Red CPL Emissions Achieved by Circularly Polarized Fluorescence Energy Transfer (CPF-ET)

**Figure 6:**
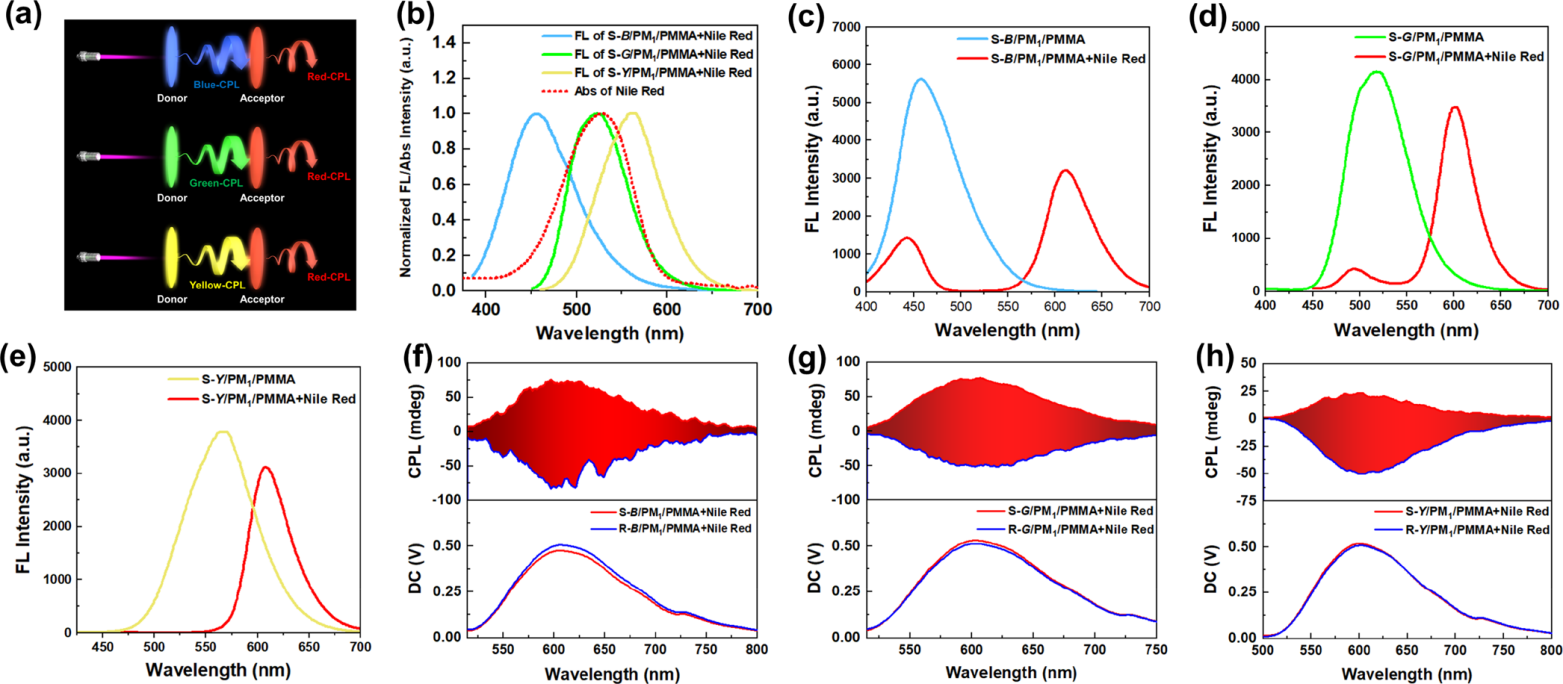
(a) Illustration of the CPF-ET process. (b) FL spectra of CPL-active films and the absorption spectrum of Nile red. (c–e) FL spectra of CPF-ET systems. (f–h) CPL spectra of CPF-ET systems (λ_ex_ = 365 nm, slit width = 3000 μm).

Organic red-light materials have received intense attention in both basic research and application research fields. However, due to the narrow band gap and largely conjugated rigid plane structure, such materials are prone to nonradiative transition, resulting in lower luminescence efficiency and even fluorescence quenching, which brings about enormous difficulty in the design and preparation of molecules.^57,58^ Therefore, how to simply and effectively prepare red CPL materials with high *g*_lum_ values is still a challenge at present. In previous studies, with circularly polarized fluorescence energy transfer (CPF-ET), we have successfully used chiral fluorescent helically substituted polyacetylene as a donor to excite a multicolor fluorophore (acceptor) to emit circularly polarized light.^42^ And different from circularly polarized fluorescence resonance energy transfer ideas reported in the literature,^38,39^ we adopted the way of radiative energy transfer^40,41^ to avoid affecting the original CPL performance and harsh assembly conditions by adding fluorophore to the donor layer. Here, we introduced a CPF-ET strategy based on the present study to achieve efficient red CPL emission, as illustrated in Figure 6a. A film containing red fluorophore (Nile red) was prepared and excited with circularly polarized fluorescence emitted from the prepared CPL-active films. That is, the CPL-active film acts as a donor and Nile red acts as an acceptor. In the CPF-ET process, the CPL-active film emits highly efficient circularly polarized fluorescence after being excited by UV light, forming a chiral excitation environment. And the acceptor can effectively absorb the dissymmetric photon energy and use it for its own transition, leading to dissymmetric red fluorescence emission (red-CPL).

After exploration, Nile red was selected as the acceptor, and its UV–vis absorption spectrum overlapped with the FL emission of the three CPL-active films in a large area (Figure 6b), satisfying the donor–acceptor energy transfer process.^37^Figure 6c–e shows that Nile red has significant absorption to FL emission of CPL-active films. Excitingly, when CPL-active films were exposed to 365 nm excitation light, the emitted multicolor CPF can effectively excite the Nile red to emit mirror-imaged red CPL at a wavelength of 600 nm (Figure 6f–h). The direction of the CPL signals for CPL-active films (donor) and Nile red (acceptor) remains consistent: S-CPL stimulated Nile red to emit S-red-CPL, and R-CPL stimulated Nile red to emit R-red-CPL. In addition, the magnitude of red-CPL is related to the original intensity of the donor’s CPL and donor–acceptor matching degree. Blue-light CPL-active films lead to the strongest CPL emission, so the corresponding red-CPL intensity is the highest, with *g*_lum_ of up to −1.1 × 10^–2^. Since the emission of green-light CPL-active films is more strongly matched with the UV–vis absorption of Nile red, even though the CPL intensity of green CPL-active films is lower than that of blue-light CPL-active films, the eventually obtained red-CPL intensity can be comparable to that of the latter, and the *g*_lum_ can also reach a 1.0 × 10^–2^ order of magnitude. The red CPL excited by the yellow-light CPL-active films has the lowest intensity, in which the *g*_lum_ is −6.6 × 10^–3^. We also characterized the CPL spectrum of the Nile red acceptor at its optimal excitation wavelength (525 nm). As shown in Figure S20, no CPL signal was detected when the acceptor film was directly excited, indicating that the CPL emission from the donor film is the key to achieving red CPL. Moreover, the Φ of Nile red in the film state was calculated to be 13.5%, and the FM of the red CPL was further calculated, as shown in Table S2. The above results show that the construction of red CPL or other CPL materials by the CPF-ET strategy has definite advantages, such as convenient and efficient preparation, without interaction between the constituting components, wide application prospects, and relatively remarkable *g*_lum_.

## Conclusions

We have prepared multicolor CPL-active films with three-pair enantiomeric chiral small molecular fluorophores with various FL emission and optically inactive helical polyacetylenes. The chiral small molecular fluorophores have strong photoluminescence properties but no CPL activity. However, with the promotion of optically inactive helical polyacetylenes, the blending of the two components achieved better amplification in chirality through a simple film-forming, self-assembly process, so that the material realized CPL emission with excellent performance from nothing, with the maximum *g*_lum_ value of up to −0.028. Besides, this study enriches the potential application of optically inactive helical polyacetylenes in polymer-based CPL materials, which can achieve the same performance level without relying on chiral polymers. More interestingly, by simple design, the CPL-active films and the film containing Nile red easily realized CPF-ET with radiative energy transfer by stacking, without the need for additional self-assembly between components. The obtained red-light CPL has a maximum *g*_lum_ of −0.011. This effective and rare strategy provides a practical idea for us to study multicolor and tunable CPL materials in the future.

## Supporting Information Available

Materials and measurements used in this study; detailed experimental procedures, including the synthesis of chiral small molecular fluorophores *B* and *G*, monomer M_1_, and polymer PM_1_, and the preparation method of CPL-active films; Figures S1–S20 and Tables S1 and S2, including NMR data of *B*, *G*, and M_1_; Raman and FT-IR spectra of PM_1_ and PM_2_; and additional FL, Abs, CD and CPL data, etc. of chiral small molecular fluorophores and CPL-active films (PDF)
