# Supplementary material for: Helical Polymer Working as a Chirality Amplifier to Generate and Modulate Multicolor Circularly Polarized Luminescence in Small Molecular Fluorophore/Polymer Composite Films
Source: ACS Cent Sci. 2023 Jun 23;9(7):1409–18. doi: 10.1021/acscentsci.3c00122 (PMC10375879; doi:10.1021/acscentsci.3c00122)
Supplement: Supplementary file 1 — oc3c00122_si_001.pdf [file oc3c00122_si_001.pdf]

Supporting Information

Helical Polymer Working as Chirality Amplifier for Generating and Modulating Multi-Color Circularly Polarized Luminescence in Small Molecular Fluorophore/Polymer Composite Films

Shuo. Ma, Biao Zhao, and Jianping Deng**

State Key Laboratory of Chemical Resource Engineering, College of materials Science and Engineering

Beijing University of Chemical Technology, Beijing 100029, China

E-mail: zhaobiao@mail.buct.edu.cn; dengjp@mail.buct.edu.cn

Experimental Section

Materials

All chemicals were purchased from TCI, Aladdin, Macklin, and Tong guang (Beijing) and used as received without further purification.

Measurements

^1H NMR and ^{13}C NMR spectra were recorded on a Bruker AV 400 spectrometer in $CDCl_3$ at room temperature using tetramethylsilane (TMS) as internal standard. Circular dichroism (CD) and UV-vis absorption spectra were measured on a Jasco-810 spectropolarimeter. Fluorescence spectra were conducted on a Hitachi F-7000 FL Spectrophotometer. Circularly polarized luminescence (CPL) was measured by JASCO CPL-200 (λ_{ex} = 365 nm, slit width = 3000 μm). Digital photographs of the emission state was taken under the radiation of a commercial LED UV-chip with a wavelength of 365 nm and a power of 45 W.

Synthesis of chiral small molecular fluorophores *B* and *G*

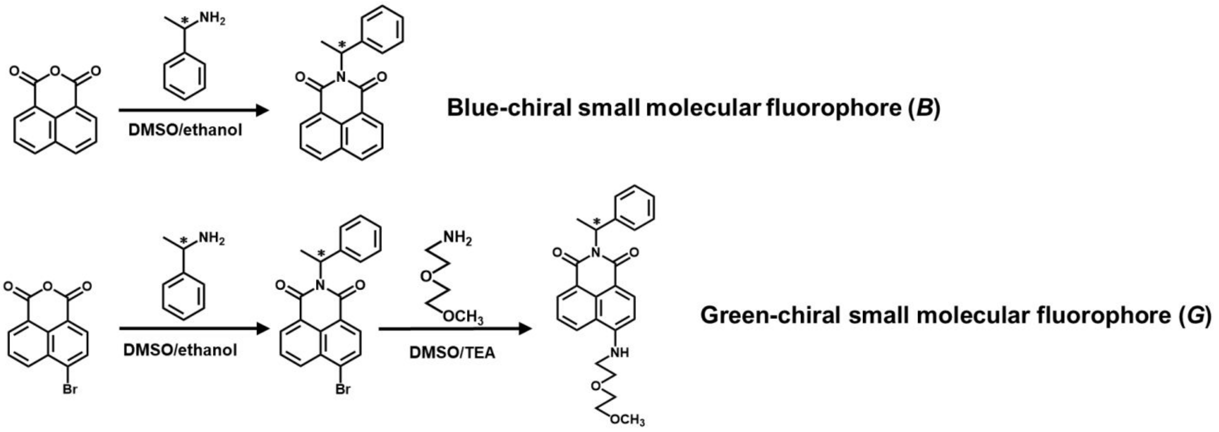

Synthesis of *B*: taking S-*B* as an example, *S*-(-)-1-phenylethylamine (*S*-PEA) (1.45 g) and 1,8-naphthalic anhydride (1.98 g) were dissolved in 50 mL mixed solvent (dimethyl sulfoxide (DMSO)/ethanol=1/1, v/v), and the mixture was stirred at 85 ℃ for 3 h. The reaction was cooled to the room temperature and the solution was washed and purified using NaOH aqueous solution, deionized water and ethanol under reduced pressure. Finally, after drying, white powder (B) was obtained (Yield = 65%).

Synthesis of *G*: taking S-*G* as an example, *S*-PEA (1.45 g) and 4-bromo-1,8-naphthalic anhydride (2.77 g) were dissolved in 50 mL mixed solvent (DMSO/ethanol=1/1, v/v), and the mixture was stirred at 85 ℃ for 3 h. The reaction was cooled to the room temperature and the solution was washed and purified using NaOH aqueous solution, deionized water and ethanol under reduced pressure. The

obtained intermediate compound and excess 2-(2-methoxyethoxy)ethanamine were dissolved in 30 mL DMSO and 1 mL triethylamine (TEA), and the mixture was reacted at 90℃ for 3 h under N_2 atmosphere. After the reaction is completed and cooled, the mixture was poured into a large amount of deionized water, filtered to obtain a yellow precipitate, neutralized with HCl dilute solution and extracted with $CHCl_2$ to obtain a crude product. After purification by column chromatography, the target product (*G*) was obtained (Yellow powder, yield = 40%).

Chiral small molecular fluorophores-*Y* is synthesized referring to reported literature.[1]

Yeild of different chiral small molecular fluorophores.

	S-*B*	R-*B*	S-*G*	R-*G*	S-*Y*	R-*Y*
Yeild (%)	65	69	40	38	51	56

Synthesis of achiral monomer M_1

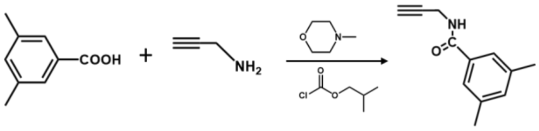

3,5-Dimethylbenzoic acid (1.5 g), *N*-methylmorpholine (0.55 mL), and isobutyl chlorocarbonate (0.65 mL) were successively added into 100 mL tetrahydrofuran (THF). After sufficient stirring, propargylamine (0.67 mL) was added dropwise to the mixture for reaction at 30 ℃ for 4 h. The reaction was filtered and diluted with ethyl acetate, and then washed with HCl dilute solution and saturated sodium bicarbonate aqueous solution in order to remove impurities. The residual water was removed by anhydrous magnesium sulfate, and a white solid powder was obtained by spin evaporation (Yield = 85%).

M_2 was synthesized according to the literature reported by our laboratory.[2]

Synthesis of achiral polymer PM_1

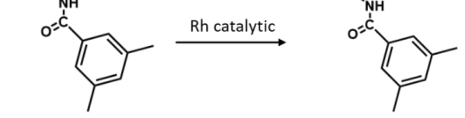

PM_1 and PM_2 were synthesized by solution polymerization. Taking PM_1 as an example: M_1 (93.5 mg) and catalyst $(nbd)Rh^+B^-(C_6H_5)_4$ (2.5 mg) were dissolved in $CHCl_3$ (5 mL), and the polymerization was carried out in N_2 atmosphere at 30 ℃ for 4 h. After reaction, the obtaining reaction solution was

poured into a large amount of *n*-hexane to precipitate out achiral polymer, and the precipitate was collected and vacuum-dried to obtain the target product (Yield = 90%).

PM_1 with with different molecular weight was prepared by changing the composition of the polymerization solvent system. Under the same polymerization process as described above, different proportions of $CHCl_3$/*n*-heptane mixed solvent were used as the polymerization reaction medium. Specifically: PM_1=$PM_{1\text{-}1}$= 5/0 (v/v); $PM_{1\text{-}2}$ = 4/1 (v/v); $PM_{1\text{-}3}$= 3/2 (v/v); $PM_{1\text{-}4}$=2/3 (v/v).

Preparation of CPL-active films

Taking S-*B*/PM_1/PMMA film as example: First, S-*B* (5 mg) and PM_1 (5 mg) were dissolved in 2 mL $CHCl_3$ to form a uniform solution. Then, PMMA (100 mg) was added into the above solution and stirred at 25 °C for 5 h. After the complete dissolution of PMMA, the mixture solution was casted onto a glass culture dish, and evaporated at 25 °C to remove the $CHCl_3$ solvent. Finally, self-supporting composite film with a uniform thickness of about 0.3 mm was obtained, as presented below.

For other CPL composite films, similar process was taken by changing different chiral small molecular fluorophores. Besides, unless otherwise specified, the film forming temperature used in the experiment is always 25 °C.

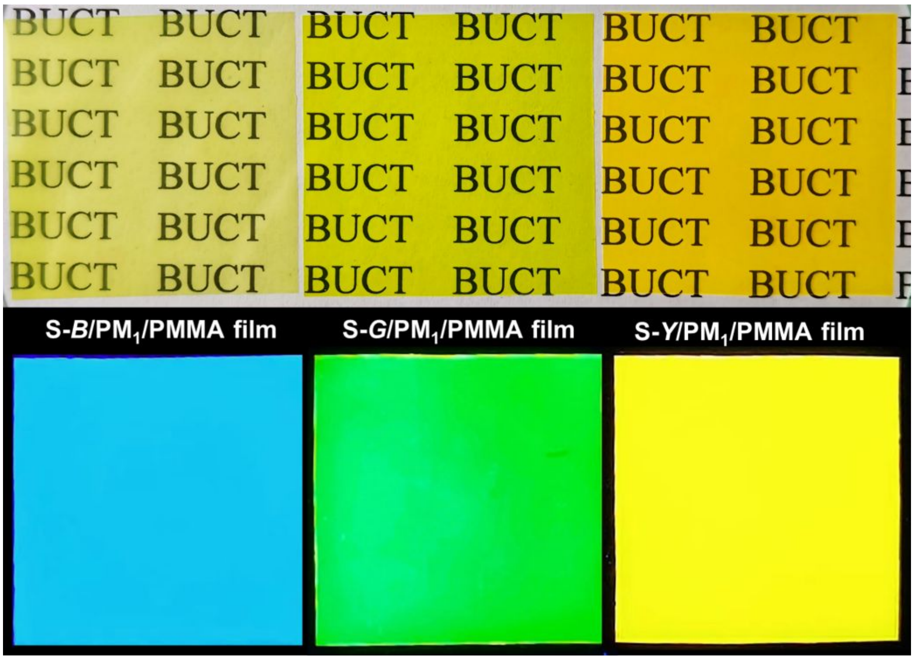

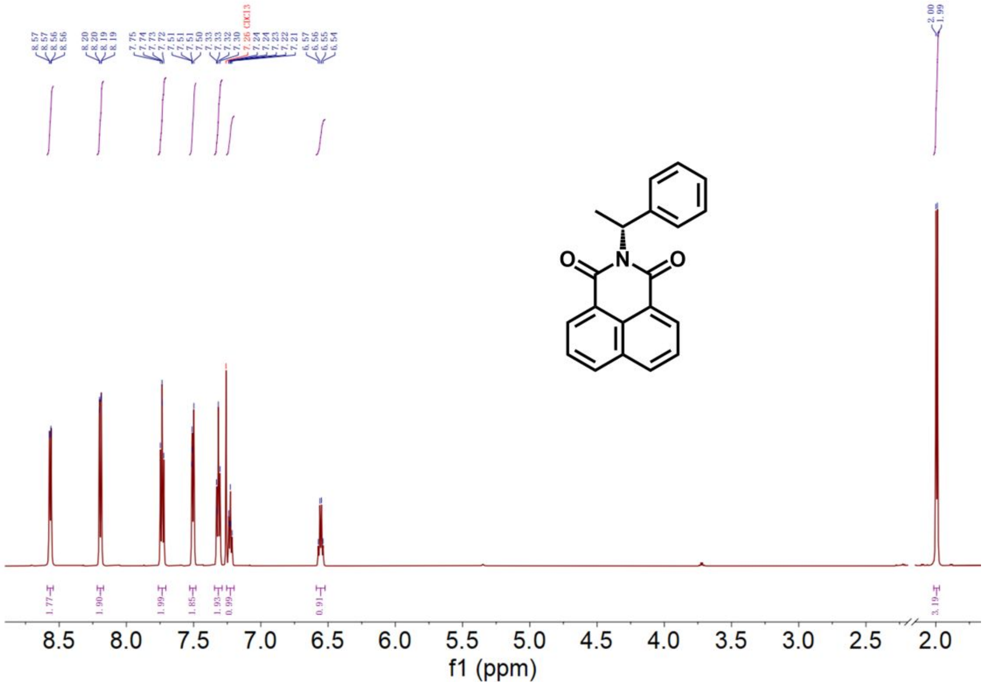

Figure S1. ^{1}H-NMR spectrum of S-*B* ($CDCl_3$, Me_4Si, 300 MHz).

8.59 – 8.54 (m, 2H), 8.19 (dd, *J* = 8.3, 1.2 Hz, 2H), 7.76 – 7.71 (m, 2H), 7.53 – 7.48 (m, 2H), 7.32 (t, *J* = 7.7 Hz, 2H), 7.23 (t, *J* = 7.6 Hz, 1H), 6.56 (q, *J* = 7.2 Hz, 1H), 1.99 (d, *J* = 7.1 Hz, 3H).

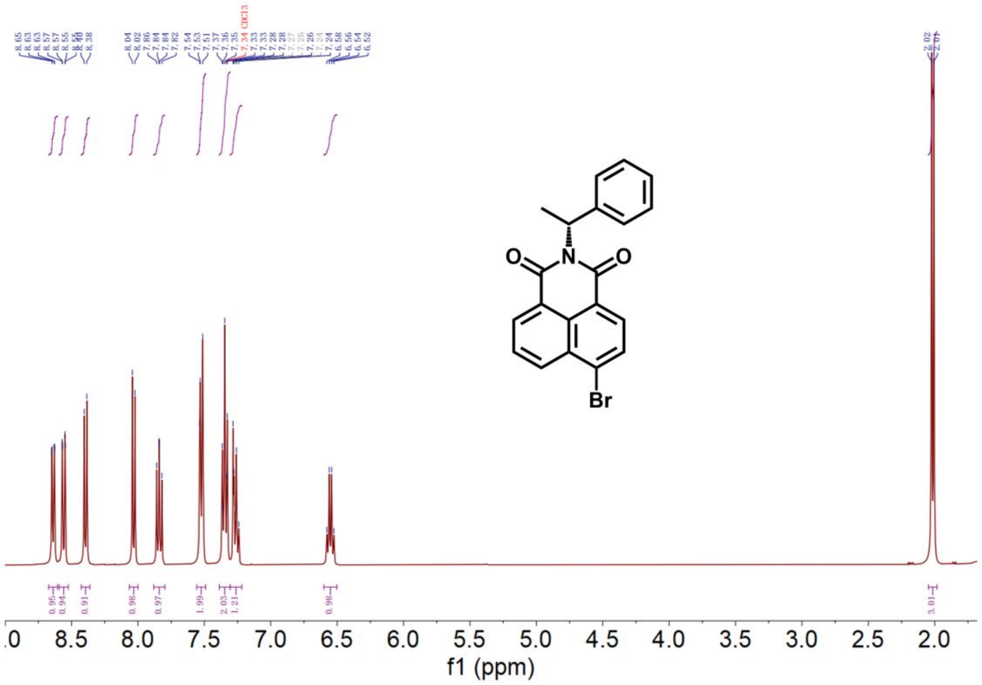

Figure S2. ^{1}H-NMR spectrum of intermediate S-*G* ($CDCl_3$, Me_4Si, 300 MHz).

8.64 (dd, *J* = 7.3, 1.1 Hz, 1H), 8.56 (dd, *J* = 8.5, 1.1 Hz, 1H), 8.39 (d, *J* = 7.9 Hz, 1H), 8.03 (d, *J* = 7.8 Hz, 1H), 7.84 (dd, *J* = 8.5, 7.3 Hz, 1H), 7.52 (d, *J* = 7.3 Hz, 2H), 7.35 (t, *J* = 7.7 Hz, 2H), 7.31 – 7.22 (m, 1H), 6.55 (q, *J* = 7.1 Hz, 1H), 2.02 (d, *J* = 7.1 Hz, 3H).

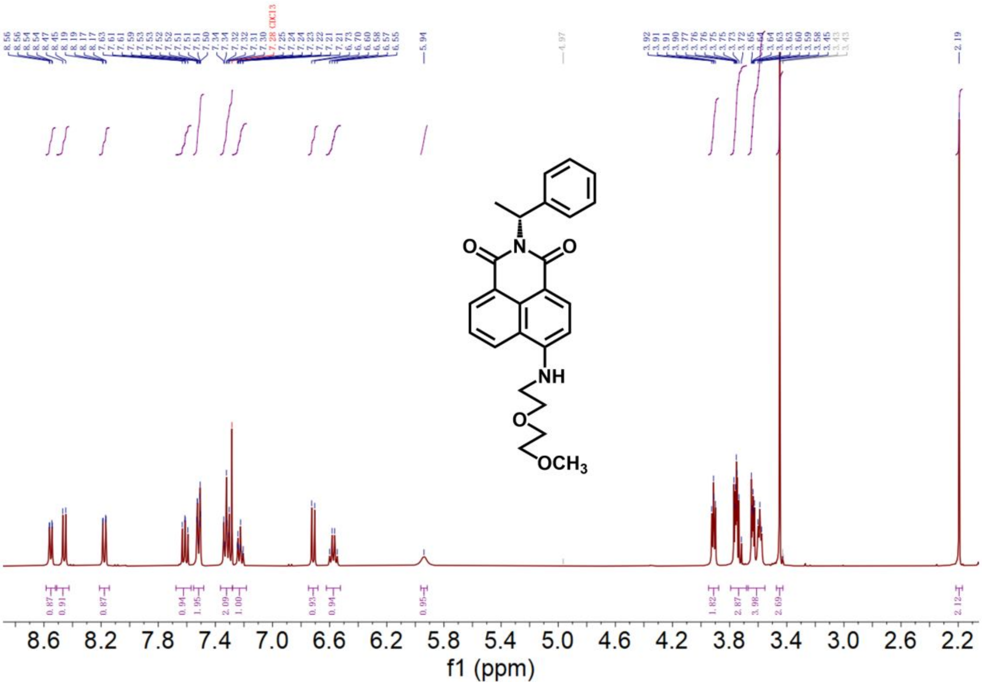

Figure S3. ^{1}H-NMR spectrum of S-*G* ($CDCl_3$, Me_4Si, 300 MHz).

8.55 (dd, *J* = 7.4, 1.0 Hz, 1H), 8.46 (d, *J* = 8.4 Hz, 1H), 8.18 (dd, *J* = 8.4, 1.1 Hz, 1H), 7.61 (dd, *J* = 8.5, 7.3 Hz, 1H), 7.55 – 7.48 (m, 2H), 7.36 – 7.28 (m, 2H), 7.28 – 7.18 (m, 1H), 6.72 (d, *J* = 8.5 Hz, 1H), 6.57 (q, *J* = 7.1 Hz, 1H), 5.94 (s, 1H), 3.91 (dd, *J* = 5.6, 4.6 Hz, 2H), 3.81 – 3.73 (m, 2H), 3.77 – 3.69 (m, 1H), 3.67 – 3.55 (m, 4H), 3.45 (s, 3H), 2.19 (s, 2H).

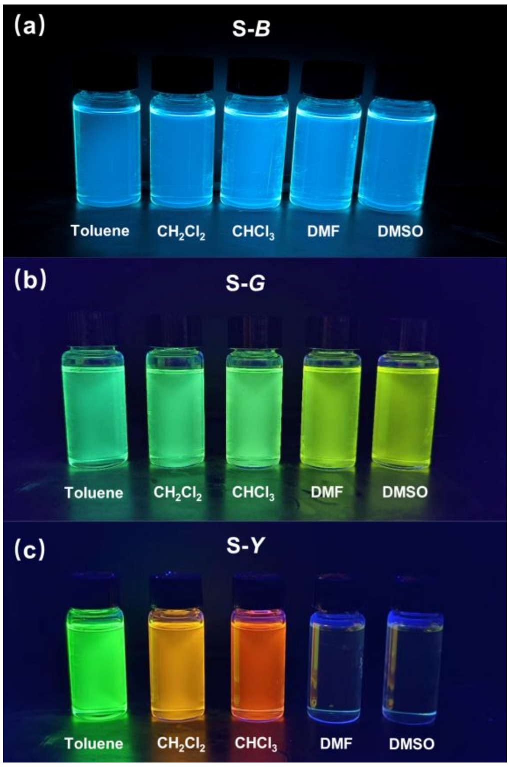

Figure S4. Photograph of S-*B*, S-*G* and S-*Y* in different solvents under UV-light (λ_{ex} = 365 nm, *c* = 0.1 mg/mL).

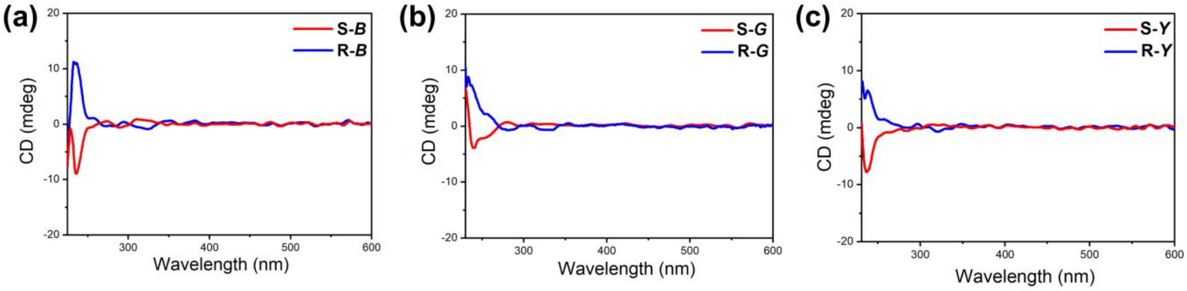

Figure S5. CD spectra of S-*B*, S-*G* and S-*Y* in $CHCl_3$ (*c* = 0.1mg/mL, pathlength = 10 mm).

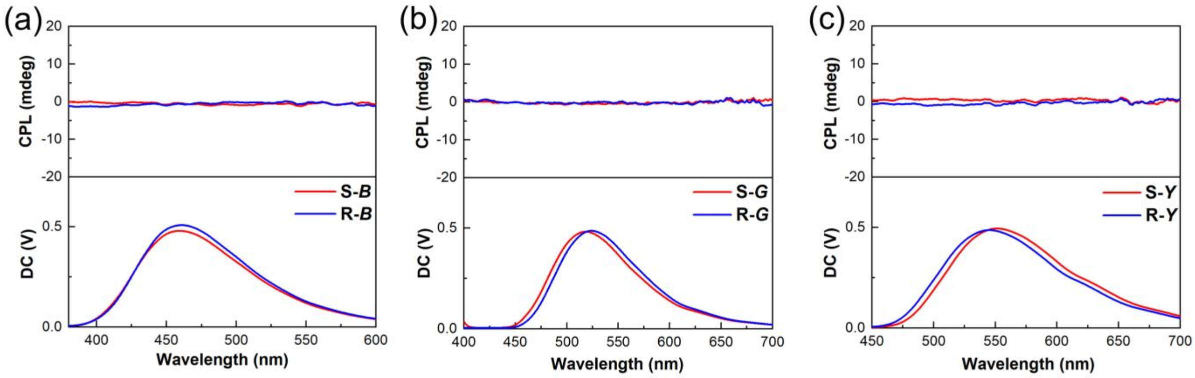

Figure S6. CPL spectra of S-*B*, S-*G* and S-*Y* in $CHCl_3$ (*c* = 0.1mg/mL, pathlength = 10 mm).

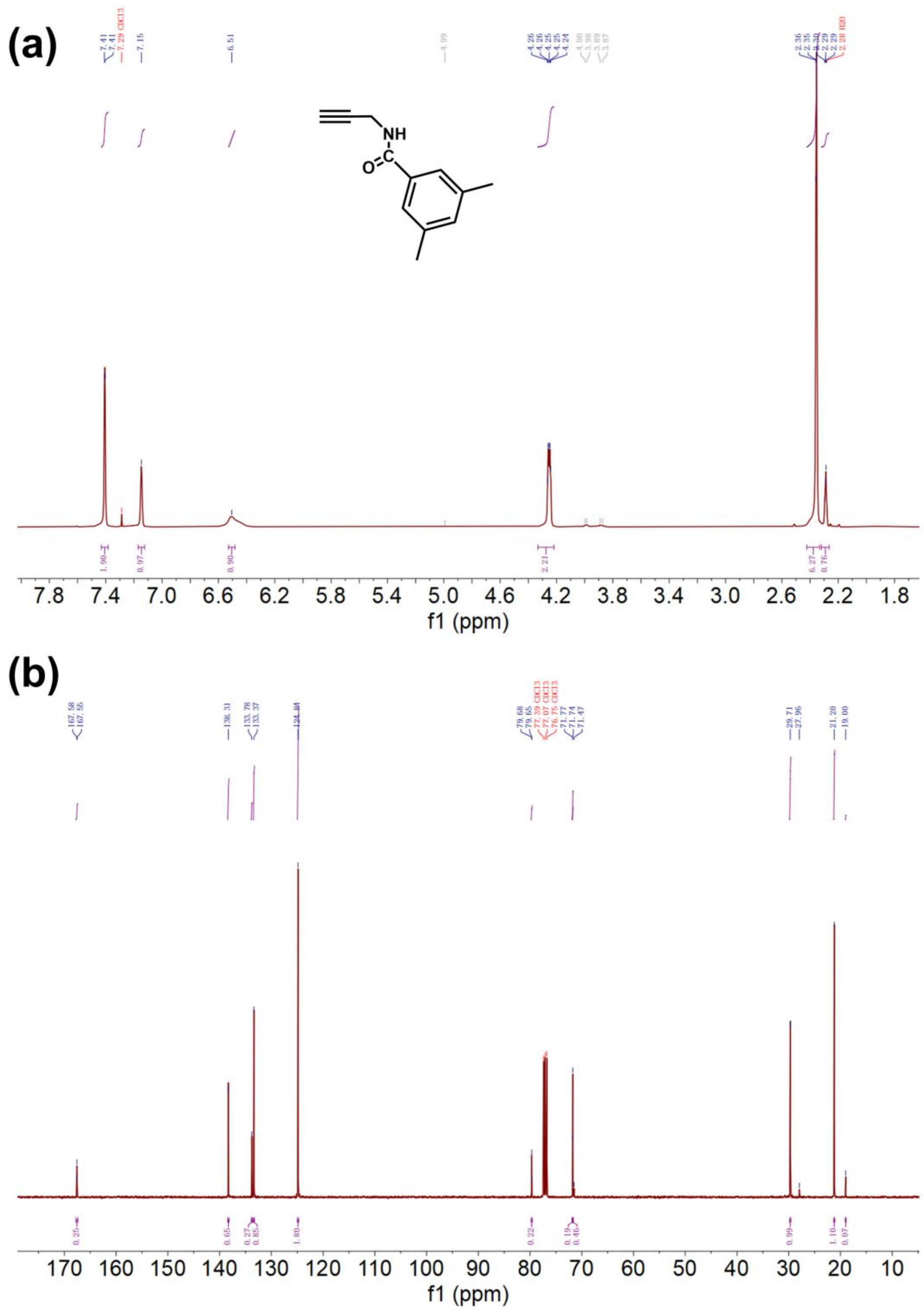

Figure S7. (a) ^{1}H-NMR and (b) ^{13}C-NMR spectra of M_1 ($CDCl_3$, Me_4Si, 300 MHz).

^{1}H-NMR: 7.41 (d, J = 1.5 Hz, 2H), 7.15 (s, 1H), 6.51 (s, 1H), 4.25 (dt, J = 4.9, 2.1 Hz, 2H), 2.36 (d, J = 2.1 Hz, 6H), 2.32 – 2.26 (m, 1H).

^{13}C-NMR: 167.58, 138.31, 133.78, 133.37, 124.84, 79.68, 71.77, 71.74, 29.71, 21.20, 19.00.

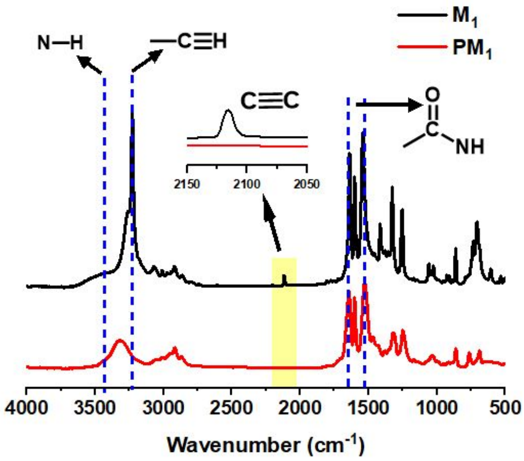

Figure S8. FT-IR spectra of M_1 and PM_1 powder in KBr tablet.

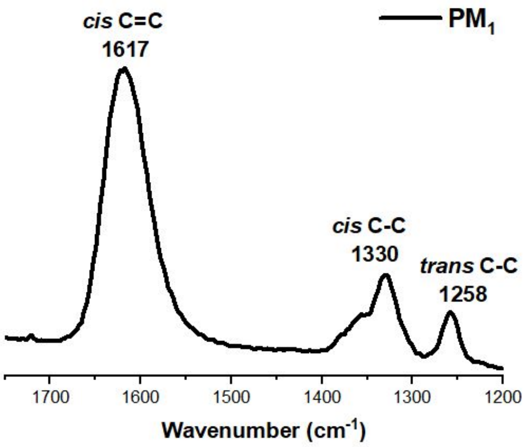

Figure S9. Raman spectrum of PM_1 powder (532 nm laser).

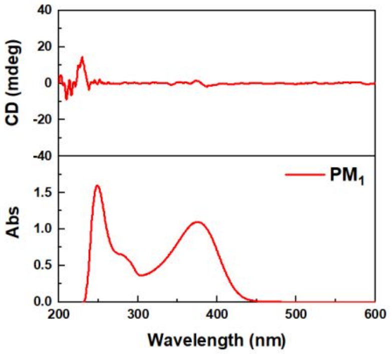

Figure S10. CD and UV-vis spectra of PM_1 ($CHCl_3$, c = 0.1mg/mL, pathlength = 10 mm).

Table S1. Absorption dissymmetry factor (g_{abs}) for chiral small molecular fluorophores and corresponding composite films.

Sample	S-*B*	R-*B*	S-*G*	S-*G*	S-*Y*	S-*Y*
Chiral small molecular fluorophore	-1.9×10^{-3}	1.9×10^{-3}	-7.8×10^{-4}	8.1×10^{-4}	-4.3×10^{-4}	$3.\times10^{-4}$
Composite films	-6.6×10^{-2}	5.5×10^{-2}	-2.7×10^{-2}	2.8×10^{-2}	-1.1×10^{-2}	1.1×10^{-3}

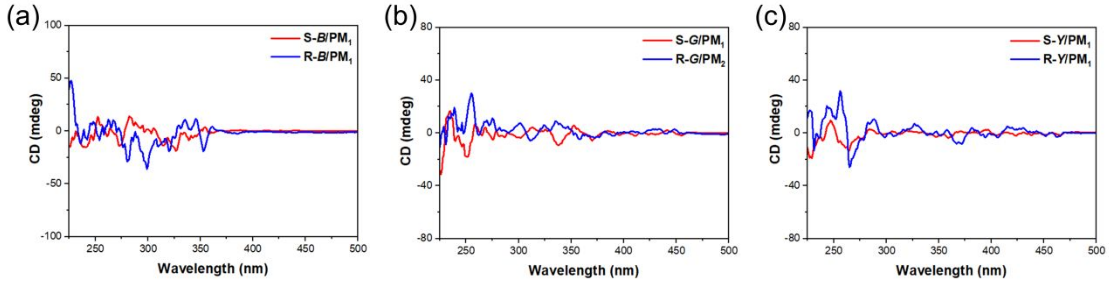

Figure S11. CPL spectra of chiral small molecular fluorophore/PM_1 mixed solutions ($CHCl_3$, $c_{small\ molecule} = c_{polymer}$ = 0.1 mg/mL, pathlength = 10 mm).

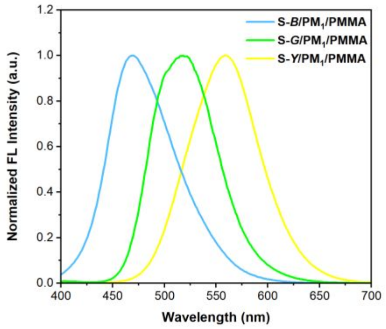

Figure S12. FL spectra of chiral small molecular fluorophores/polymer composite films (λ_{ex} = 365 nm).

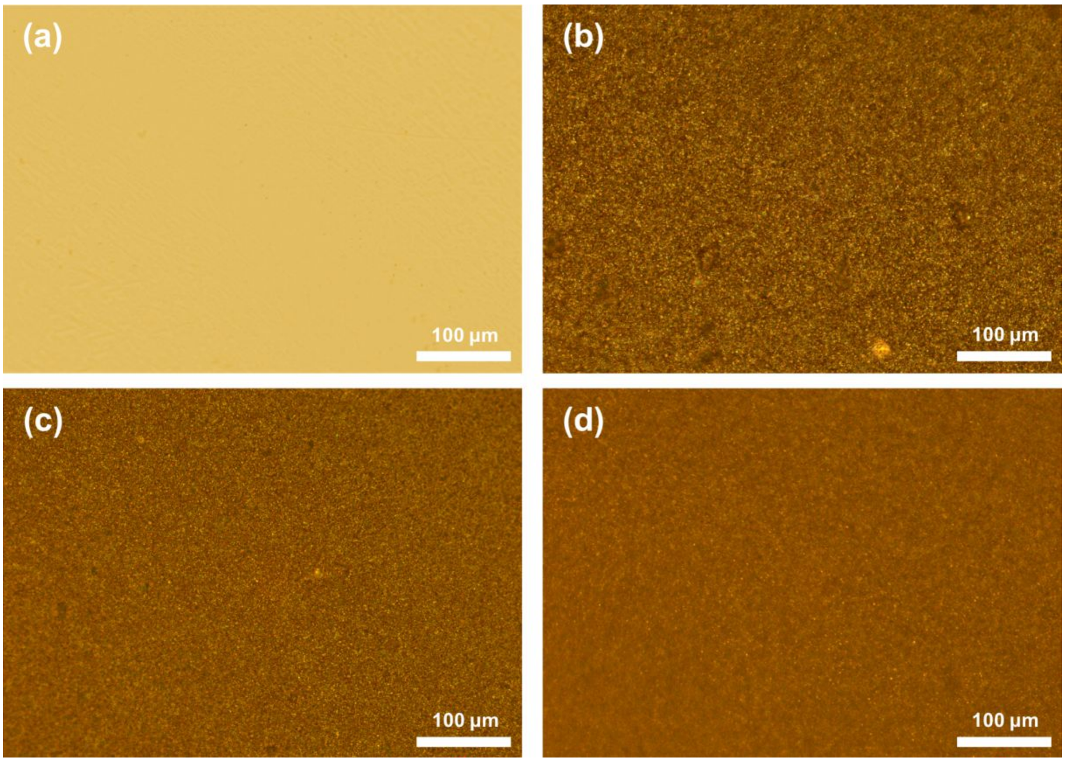

Figure S13. Optical microscope images of (a) PMMA film, (b) S-*B*/PM_1/PMMA film, (c) S-*G*/PM_1/PMMA film and (d) S-*Y*/PM_1/PMMA film.

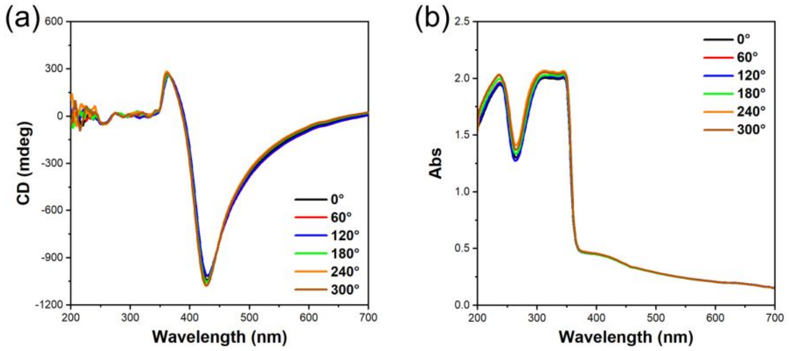

Figure S14. (a) CD and (b) UV-vis spectra of S-*B*/PM_1/PMMA film measured under different rotation angles.

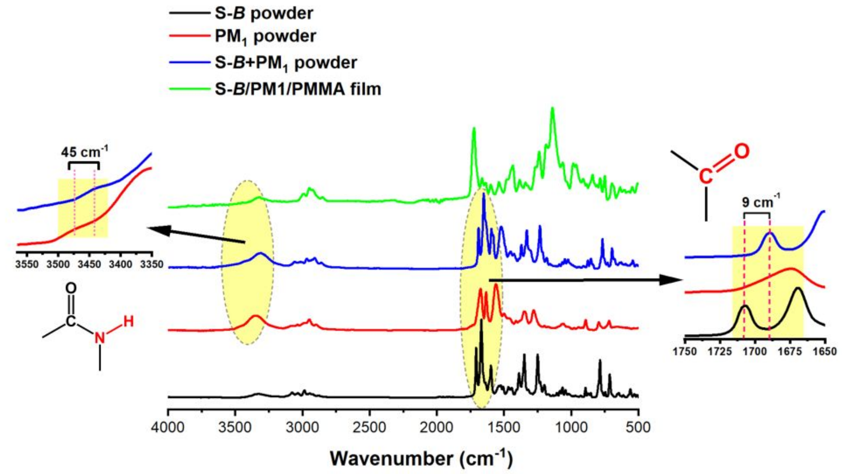

Figure S15. FT-IR spectra of S-*B*, PM_1, S-*B*+PM_1 powder in KBr tablet, and S-*B*/PM_1/PMMA film.

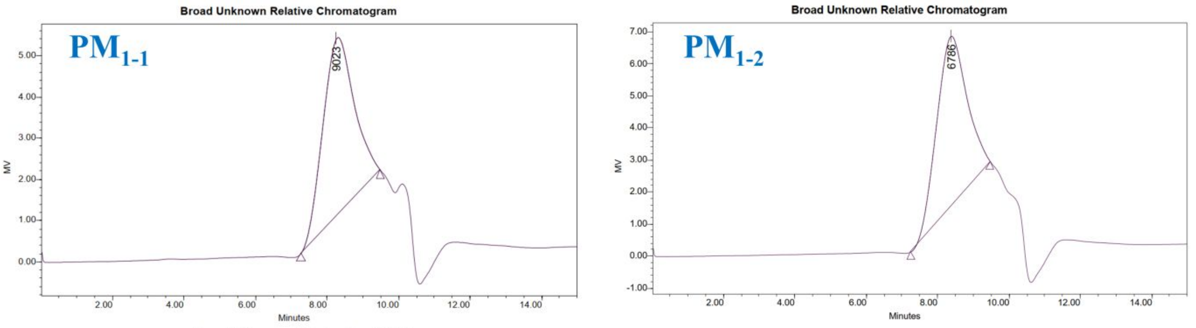

Broad Unknown Relative Peak Table (PM_{1-1})

	Distribution Name	Mn (Daltons)	Mw (Daltons)	MP (Daltons)	Mz (Daltons)	Mz+1 (Daltons)	Polydispersity	Mz/Mw	Mz+1/Mw
1		5178	10965	9023	19992	30118	2.117645	1.823295	2.746813

Broad Unknown Relative Peak Table (PM_{1-2})

	Distribution Name	Mn (Daltons)	Mw (Daltons)	MP (Daltons)	Mz (Daltons)	Mz+1 (Daltons)	Polydispersity	Mz/Mw	Mz+1/Mw
1		4671	9217	6786	16344	24494	1.973195	1.773274	2.657480

Broad Unknown Relative Peak Table (PM_{1-3})

	Distribution Name	Mn (Daltons)	Mw (Daltons)	MP (Daltons)	Mz (Daltons)	Mz+1 (Daltons)	Polydispersity	Mz/Mw	Mz+1/Mw
1		4067	8330	6917	14580	21803	2.048202	1.750206	2.617306

Broad Unknown Relative Peak Table (PM_{1-4})

	Distribution Name	Mn (Daltons)	Mw (Daltons)	MP (Daltons)	Mz (Daltons)	Mz+1 (Daltons)	Polydispersity	Mz/Mw	Mz+1/Mw
1		3477	8053	6578	15379	23976	2.316242	1.909857	2.977463

Figure S16. GPC data of PM_1 with different molecule weight.

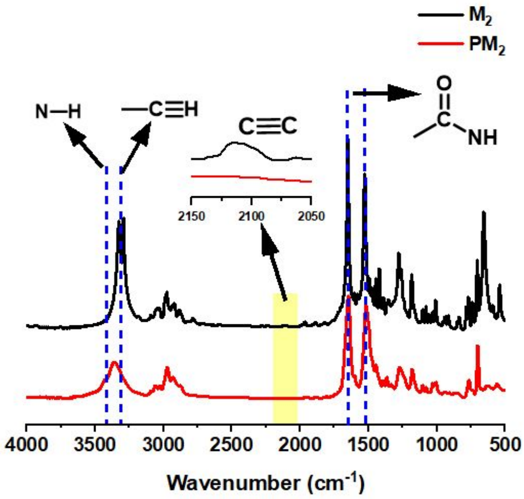

Figure S17. FT-IR spectra of M_2 and PM_2 powder in KBr tablet.

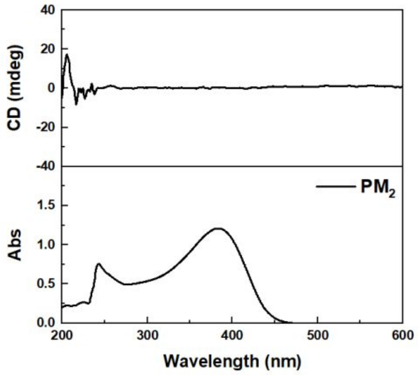

Figure S18. CD and UV-vis spectra of PM_2 ($CHCl_3$, c = 0.1 mg/mL, pathlength = 10 mm).

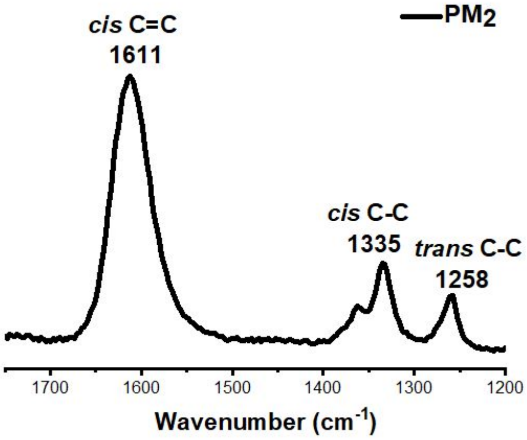

Figure S19. Raman spectrum of PM_2 powder (532 nm laser).

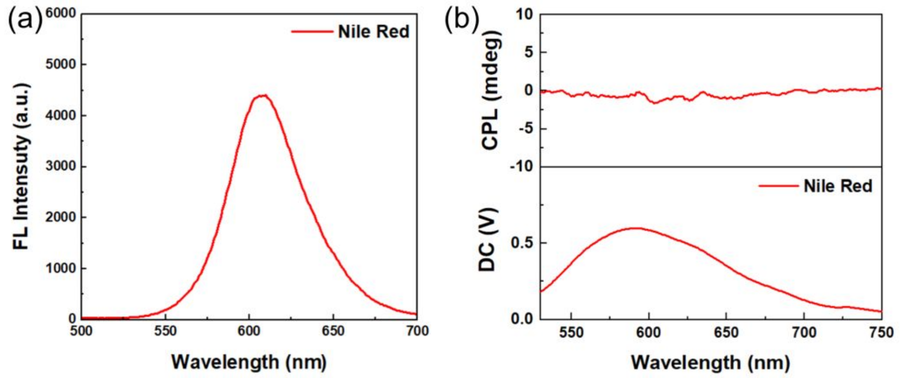

9

Figure S20. (a) FL and (b) CPL spectra of Nile Red (accepter) in PMMA film (λ_{ex} = 525 nm).

Table S2. Summary of the CPL data in the red-CPL systems.

	S-*B*/PM_1/PMMA + Nile red	R-*B*/PM_1/PMMA + Nile red	S-G/PM_1/PMMA + Nile red	R-G/PM_1/PMMA + Nile red	S-Y/PM_1/PMMA + Nile red	R-Y/PM_1/PMMA + Nile red
Wavelength	602 nm	608 nm	607 nm	603 nm	602 nm	602 nm
g_{lum}	1.0×10^{-2}	-1.1×10^{-2}	1.0×10^{-2}	-6.7×10^{-3}	3.1×10^{-3}	-6.6×10^{-3}
FM	1.4×10^{-3}	-1.5×10^{-3}	1.4×10^{-3}	-9.0×10^{-4}	4.2×10^{-4}	-8.9×10^{-4}

Reference

(1) Li, Y.; Yao, K.; Chen, Y.; Quan, Y.; Cheng, Y. Full-Color and White Circularly Polarized Luminescence Promoted by Liquid Crystal Self-Assembly Containing Chiral Naphthalimide Dyes. *Adv. Opt. Mater.* **2021,** *9* (20), 2100961.

(2) Lin, J.; Huang, H.; Wang, M.; Deng, J. Optically active hollow nanoparticles constructed by chirality helical substituted polyacetylene. *Polym. Chem.*, **2016**, *7* (8), 1675–1681.
